# A Porous Fluoride-Substituted Bovine-Derived Hydroxyapatite Scaffold Constructed for Applications in Bone Tissue Regeneration

**DOI:** 10.3390/ma17051107

**Published:** 2024-02-28

**Authors:** Jithendra Ratnayake, Maree Gould, Niranjan Ramesh, Michael Mucalo, George J. Dias

**Affiliations:** 1 Department of Oral Science, Faculty of Dentistry, University of Otago, Dunedin 9016, New Zealand; maree.gould@otago.ac.nz; 2 Department of Anatomy, School of Biomedical Sciences, University of Otago, Dunedin 9054, New Zealand; niranjan.ramesh@otago.ac.nz (N.R.); george.dias@otago.ac.nz (G.J.D.); 3 School of Science, University of Waikato, Hamilton 3216, New Zealand; michael.mucalo@waikato.ac.nz

**Keywords:** hydroxyapatite, bovine hydroxyapatite, ionic substitutions, fluorapatite, characterisation, biocompatibility

## Abstract

Hydroxyapatite is widely used in bone implantation because of its similar mineral composition to natural bone, allowing it to serve as a biocompatible osteoconductive support. A bovine-derived hydroxyapatite (BHA) scaffold was developed through an array of defatting and deproteinization procedures. The BHA scaffold was substituted with fluoride ions using a modified sol-gel method to produce a bovine-derived fluorapatite (BFA) scaffold. Fourier-transform infrared spectroscopy and X-ray diffraction analysis showed that fluoride ions were successfully substituted into the BHA lattice. According to energy dispersive X-ray analysis, the main inorganic phases contained calcium and phosphorus with a fluoride ratio of ~1–2 wt%. Scanning electron microscopy presented a natural microporous architecture for the BFA scaffold with pore sizes ranging from ~200–600 μm. The BHA scaffold was chemically stable and showed sustained degradation in simulated-body fluid. Young’s modulus and yield strength were superior in the BFA scaffold to BHA. In vitro cell culture studies showed that the BFA was biocompatible, supporting the proliferative growth of Saos-2 osteoblast cells and exhibiting osteoinductive features. This unique technique of producing hydroxyapatite from bovine bone with the intent of producing high performance biomedically targeted materials could be used to improve bone repair.

## 1. Introduction

The human body is incapable of regenerating large critical bone defects, and, therefore, hard tissue engineering answers that need, but this requires a material that is both robust and biocompatible for successful bone regeneration. Bone loss can occur as a result of fractures, cancer or conditions such as osteoporosis. Treatment costs involved in addressing bone trauma add to the financial burden of healthcare systems. Traditionally, bone damage has been addressed by either the use of autografts or allografts. However, the above practices present significant complications such as limited availability, donor site morbidity, disease transmission and immune rejections [1,2].The above shortcomings have channeled research into new directions in the quest for alternative bone substitutes leading to the emergence of biomaterials. Bone tissue engineering was one of the several fields that stood to benefit from the advent of biomaterials research. Xenograft bone substitutes and other natural & synthetic biomaterials provide an efficient and cost-effective alternative to address bone defects beyond critical size. An ideal scaffold for bone tissue engineering applications should possess a 3D dimension for cell attachment and proliferation, bioactive molecules such as growth factors and cytokines which can module activity of a cell and a substrate for cell differentiation [2].

The apatite lattice structure of hydroxyapatite is represented by (A_10_(BO_4_)_6_X_2_) where A, B and X denotes calcium (Ca^2+^), phosphate (PO_4_^3−^) and OH^−^ respectively [3]. Hydroxyapatite is a bioactive, non-toxic and osteoconductive material, therefore, it is commonly used as a coating material for dental implants and to repair bone defects in maxillofacial and orthopaedic surgery [4,5]

HA can be produced synthetically or from natural resources. Biologically derived HA from sources such as bovine bone is of strong interest due to its structural similarity to human bone [3,6]. Bovine cancellous bone possesses the correct trabecular interconnected porous architecture, which assists in osteogenesis and angiogenesis [6,7]. Trace ions such as carbonate, magnesium, sodium and chloride are found in sintered bovine bone, which is similar to the mineral content of human bones and teeth. These ions play a vital role in bone metabolism and osteointegration [6,8]. Due to the low cost of the raw material, the presence of trace elements and structural properties such as interconnected porous architecture, bovine bone-derived hydroxyapatite (BHA) is advantageous over synthetic hydroxyapatite as a bone graft substitute material [6,7]. Nevertheless, hydroxyapatite is brittle and has a low fracture toughness, limiting its applications to only non-load-bearing conditions. 

The lattice structure of hydroxyapatite is favorable to ionic substitutions [3,9]. To resemble the calcium-deficient nature of natural hydroxyapatite in human bone and teeth, ionic substituents have been incorporated into synthetic HA. When the calcium site in HA is partially replaced with ions such as Mg^2+^, Zn^2+^ or Ag^+^, cationic substitutions occur [9]. There are two types of anionic substitutions. An ‘A type’ substitution occurs when larger halides such as chloride or fluoride (Cl^−^ or F^−^) ion replaces the hydroxyl ion. ‘B type’ substitution occurs when silicate (SiO_4_^4−^) or carbonate functional groups replace the phosphate functional groups in the HA lattice [3,10]. 

In the past, various chemical methods successfully synthesized fluorapatite (FA) and fluorohydroxyapatite (F-partially substituted hydroxyapatite/FHA). Several studies have shown that fluoride ions stimulate osteoblasts, thereby enhancing cell proliferation and differentiation, leading to superior biocompatibility properties [11,12]. In addition, substituting fluoride ions into HA increases mechanical properties and crystallinity and decreases solubility [12,13]. However, HA sourced from bovine bone is not susceptible to ionic substitutions due to its highly ordered crystal structure [14]. Therefore, this study aimed to substitute fluoride ions into bovine-derived hydroxyapatite to create a bovine-derived fluorapatite (BFA) scaffold while maintaining the interconnected porous network, facilitating angiogenesis and osteogenic cell migration.

## 2. Materials and Methods

### 2.1. Preparation of Bovine Fluorapatite (BFA)

Bovine-derived hydroxyapatite (BHA) was produced from bovine bone using a defatting and deproteinising procedure involving pressure cooking, sodium hydroxide treatment, microwave processing, subcritical water extraction and low-temperature sintering [7,15]. A modified sol-gel process was used to incorporate fluoride ions into BHA to produce bovine fluorapatite (BFA) [16]. The sol-gel method described below was used to synthesise the F sol-gel. The (Ca)/(P) mole ratio and the (P)/(F) mole ratio were maintained at 1.677 and 3. 

**Figure 1:**
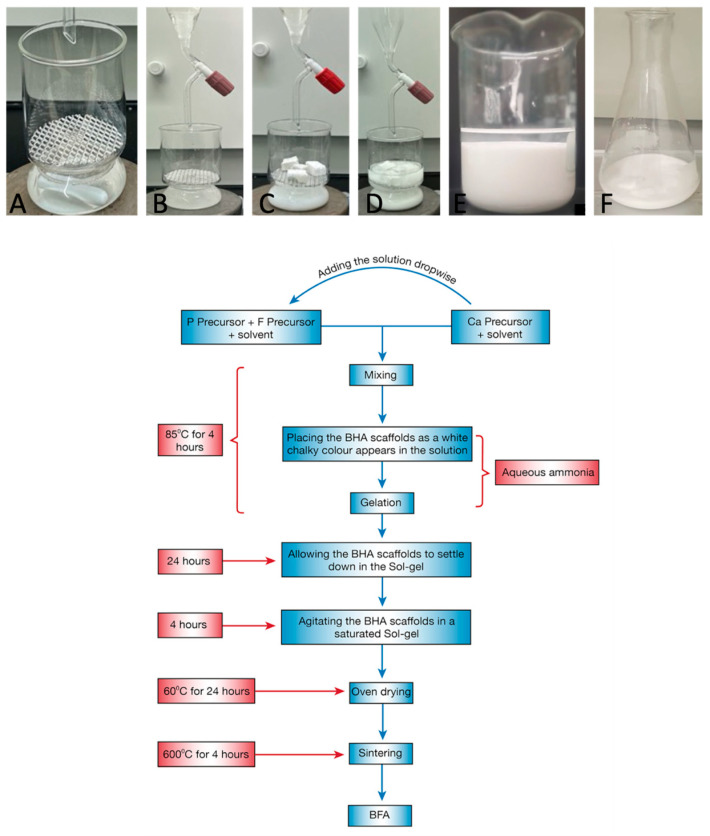
The sol-gel process to substitute fluoride ions into BHA. (**A**) Custom-made beaker consisting of a removable wire mesh and the P-F solution. (**B**) Addition of the Ca solution dropwise using a glass dropping funnel. (**C**) Placement of the bovine bone cubes on the wire mesh as a white chalk colour appears. (**D**) Stirring the solution until gel formation. (**E**) Allowing the gel to settle at room temperature for 2 h. (**F**) Placing the bone cubes in the saturated sol-gel.

Controlled amounts of ammonium hydrogen phosphate ((NH_4_)_2_HPO_4_; Sigma Aldrich, Auckland, New Zealand) and ammonium fluoride (NH_4_F; Sigma Aldrich NZ) (Table 1) were first dissolved in de-ionised water with vigorous stirring for 15 min, and the phosphorus and fluoride (P-F) solution was transferred into a custom-made beaker (Figure 1A). The calcium solution was prepared by dissolving a stoichiometric amount of calcium nitrate tetrahydrate (Ca(NO_3_)_2_.4H_2_O; Sigma Aldrich NZ) (Table 1) in de-ionised water for 30 min with a magnetic stirrer. The calcium-containing solution was added dropwise to the P-F solution with the aid of a glass-dropping funnel (Figure 1B) until a white chalky colour appeared. Then 5–6 processed BHA cubes were placed immediately onto the wire mesh in the custom-made beaker (Figure 1C). The remaining Ca(NO_3_)_2_.4H_2_O solution was added dropwise directly onto the processed bovine bones under mild stirring/agitating conditions. To maintain a pH above 10.5 during the experiment, aqueous ammonia (Sigma Aldrich, NZ) was added to the solution. The combined solution was stirred for 4 h until the formation of a gel (Figure 1D), and the gel was allowed to settle for 23 h. The bone cubes were removed from the gel, and the solution containing the gel was transferred to a conical flask and allowed to settle for a further 2 h (Figure 1E). Once the gel was settled and supernatant was visible, the supernatant was removed and the bone cubes were placed in the saturated F sol-gel. An orbital shaker was used to agitate the bone cubes in the saturated sol-gel. Finally, the bone cubes were removed and dried in a domestic oven at 60 °C for 24 h prior to sintering at 300 °C for 4 h. 

### 2.2. Characterisation

A Fourier Transform Infrared Spectrometer (FTIR) (Perkin Elmer, Darmstadt, Germany) operating in the mid-infra-red region over a wavelength range of 400–4000 cm^−1^ was used to determine the functional groups of both BHA and BFA. The phase composition of the BHA and BFA was determined on a PANalytical X¹Pert PRO MPD System (PW3040/60) X-ray diffractometer using Cu-Kα radiation (K-Alpha1 = 1.54060[Å] [4], K-Alpha2 = 1.54443[Å]). The generator was set to 40 kV and 40 mA. The XRD data for each sample was collected in the region 10° < 2θ < 70° with a step size of 0.020 and a step time of 2.5 s. The BFA patterns were identified by comparing the diffraction patterns of fluorapatite (FA) (JPDS pattern 01-080-3032) in the PANalytical XPert Highscore database. The structural details, including the pore diameter, and elemental chemical composition, were studied using scanning electron microscopy (SEM) (Oxford JOEL 2300, Tokyo, Japan) equipped with an energy-dispersive X-ray analyser (EDX, Cambridge, UK) working at 15 kV and 15 mA. In addition, inductively coupled plasma mass spectrometry (ICP-MS; Agilent 7500cs quadrupole analyser) was used to analyse the chemical composition and to measure the calcium to phosphorous ratio of the resulting BHA and BFA scaffolds. The data were compared with EDX analysis. Micro-computed Tomography (µCT) was performed on the BFA scaffold to quantify the 3D structure and porosity using a SkyScan 1172 high-resolution µCT scanner (Bruker, Kontuch, The Netherlands). The prepared BFA scaffold’s thermal stability and decomposition were studied using a TGA analyser (Q50, TA Belgium) from 20–1000 °C at a continuous heating rate of 10 °C.min^−1^ in an inert N_2_ atmosphere.

### 2.3. Chemical Stability and Degradation in Simulated Body Fluid

BFA scaffold in vitro chemical stability and degradation were assessed using a simulated body fluid (SBF) solution [7,17]. BFA scaffolds (n = 3, size: 10 mm^3^) were incubated in Falcon tubes containing 10 mL of SBF solution at 37 °C for 1, 3, 5, 14, 21 and 28 days. A pH conductometer (Ionode, New Zealand) was used to measure the pH value of the desired time point. To measure the degradation, the scaffolds were removed from the SBF solution and rinsed gently under de-ionised water before oven drying for 24 h at 60 °C. The degradation of the scaffolds was measured using the following formula.

W_L_ = (W_0_ − W_1_)/W_1_ × 100%

where W_0_ and W_1_ represent the weights of the sample before and after immersion. 

### 2.4. Mechanical Properties

The mechanical properties of the BFA and BHA scaffolds (n = 20; 10 cm × 10 cm × 10 cm) were measured using an Instron compression tester according to the protocol established in our previous study [6]. To determine the statistical significance differences (*p* < 0.05) in the mechanical properties, a two-tail Student’s *t*-test was performed on the mechanical properties of each material.

### 2.5. Cell Culture

Human osteosarcoma cells (Saos-2) (ATCC^®^ HTB-85™, American Type Culture Collection (ATCC), Manasas, VI, USA) were used to evaluate the biocompatibility of the BHA and BFA scaffolds. Saos-2 cells were cultured in a 25 cm^2^ cell culture flasks (Invitrogen, New Zealand) in a humidified incubator operating at 37°C with 5% CO_2_ using minimum essential medium alpha (MEM-a; Invitrogen, New Zealand) supplemented with 10% foetal bovine serum (FBS; Thermo Fisher Scientific, Auckland, New Zealand) and 1% penicillin–streptomycin antibiotics (10,000 units/10 mL penicillin, 10 µg/10 mL streptomycin, 25 µg/mL amphotericin B) (Life Technologies, Auckland, New Zealand) [18]. The differentiation medium was prepared by the addition of 10 nM dexamethasone, 5 mM β-glycerophosphate (β-GP) and 100 μM L-ascorbic acid 2 –phosphate supplements to the Saos-2 growth medium (S-GM). The osteogenic medium was prepared freshly every time before each experiment. The BHA and BFA scaffolds were formed into discs using an 8 mm biopsy punch (8 mm × 3 mm) and sterilised by immersion in 70% ethanol for 30 min under UV radiation, followed by rinsing with phosphate-buffered saline (PBS) for 2 min. Finally, the sterilised scaffolds were equilibrated overnight in 1 mL of culture medium in a humidified incubator. Cells were seeded directly onto each scaffold at a density of 6 × 10^3^ cells/scaffold once the cells reached 70–80% confluency. The cells were incubated for 1 h to allow cells to adhere before medium was added. The culture medium was replaced daily. All experiments were carried out in triplicate and repeated three times (n = 9).

#### 2.5.1. Cell Viability/Cytotoxicity Assay

The cell cytotoxicity was determined according to the manufacturer’s instructions using the LIVE/DEAD^®^ cytotoxicity kit for mammalian cells (Life Technologies, Auckland, New Zealand). Fluorescent visualisation was performed using a fluorescence microscope.

#### 2.5.2. Cell Proliferation Assay

The cell proliferation of Saos-2 cells on the scaffolds was determined using the MTS [3-(4,5-dimethylthiazol-2-yl)—5-(3-carboxymethoxyphenyl)—2-(4-sulphonyl)—2H-tetrazolium] assay using the manufacturer’s standard protocol. After 24, 48 and 72 h, absorbance was recorded using a spectrophotometer (Synergy 2 Multi-Mode Microplate Reader, Biotek^®^, Winooski, CA, USA) at the wavelength of 490 nm. 

#### 2.5.3. Alkaline Phosphatase (ALP) Activity

ALP activity was assessed using the protocol established in our previous study [4]. To assess the ALP activity, 20 × 10^3^ cells were seeded on the scaffolds and the cells were fed with the differentiation medium. The ALP activity was assayed at 1, 3, 7 and 14 days using the SensoLyte^®^ pNPP Alkaline Phosphatase Assay kit (AnaSpec/EGT Group, Fremont, CA, USA) according to the manufacturer’s instructions. The ALP concentration was measured at 410 nm using a spectrophotometer (Synergy 2 Multi-Mode Microplate Reader, Biotek) and the ALP activity was calculated from the standard curve. ALP activity was normalized to total protein content as determined by a Qubit fluorometer (Life Technologies, Carlsbad, CA, USA). Therefore, the ALP activity was expressed as units per microgram (μg) of protein. 

#### 2.5.4. Immunohistochemical Analysis

The osteoinductivity of the scaffold was assessed using immunofluorescence to observe the expression of a specific bone marker protein “*osteonectin*”. Cells were seeded at a cell density of 20 × 10^3^ cells/scaffold and the cell-scaffold constructs were fed with the differentiation medium. At the end of the 14-day culture period, cell-scaffolds were fixed with 10% neutral buffered formalin for 4 h and rinsed twice with PBS. Methanolic peroxo-block was added to each scaffold for 10 min and rinsed with PBS. Subsequently, the scaffolds were blocked with 10% donkey serum for 10 min for non-specific blocking and then incubated with the primary antibody *mouse anti-osteonectin* (1:500 dilution) (Duolink, Sigma Aldrich, Auckland, New Zealand) for 24 h at 4 °C. A negative control scaffold using normal mouse IgG (Dako) was always used. The following day, the scaffolds were washed with phosphate buffered saline and incubated for two hours at RT with the Alexa fluor donkey anti-mouse 488 secondary antibody (1:200 dilution). The excitation/emission of Alexa fluor donkey anti-mouse 488 was 495/519 nm. The scaffold was counterstained with 4’,6-diamidino-2-phenylindole (DAPI) (Duolink, Sigma Aldrich, Auckland, New Zealand) to visualize the nucleus of the cells. Finally, the scaffolds were transferred onto a glass coverslip and were visualized using a Zeiss LSM510 upright confocal laser-scanning microscope (Carl-Zeiss-Strasse, Oberkochen, Germany), and images were captured using Q-capture 3.1.2 software.

#### 2.5.5. Statistical Analysis

PRISM (GraphPad Prism 6, version 9.0, Boston, MA, USA) software was used to perform the statistical analysis. Error bars were reported as ± standard error of the mean (S.E.M.). A one-way analysis of variance ANOVA was used to determine statistically significant differences. If differences were detected, multiple comparisons were made using Tukey’s multiple comparison tests at a confidence level of 95% (*p* < 0.05). 

## 3. Results

### 3.1. Characterization

#### 3.1.1. Fourier Transform Infrared (FTIR) Spectroscopy

**Figure 2:**
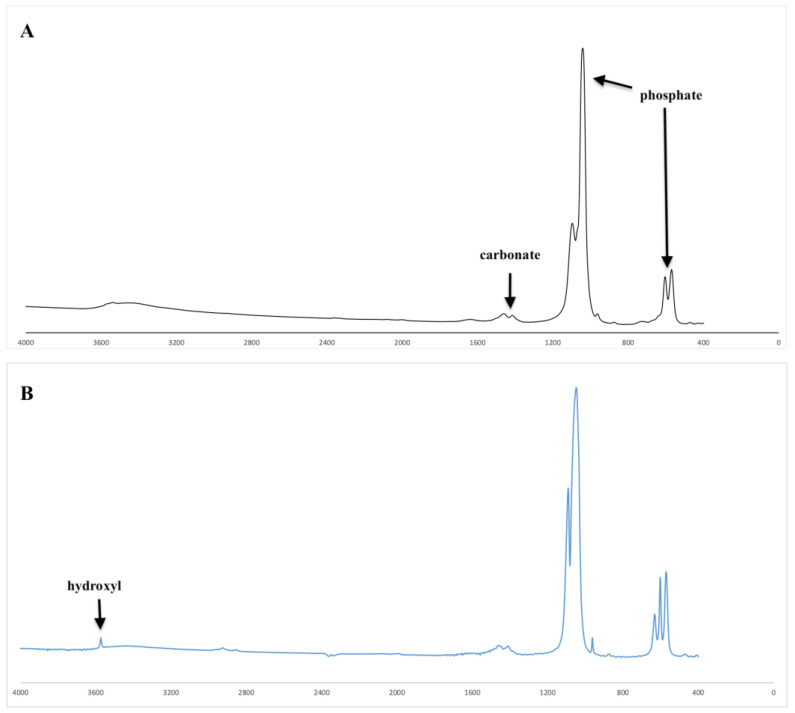
Fourier transform infra-red spectra of BFA (**A**) and BHA (**B**).

The FTIR spectra for BFA (A) and BHA (B) are presented in Figure 2. The FTIR spectra for both BHA and BFA samples showed characteristic phosphate and carbonate peaks associated with hydroxyapatite [7]. However, the hydroxyl peak due to lattice OH in the hydroxyapatite, which was observed at 3571 cm^−1^ in the BHA spectrum, was absent in the BFA spectrum.

#### 3.1.2. X-ray Diffraction (XRD) Analysis

**Figure 3:**
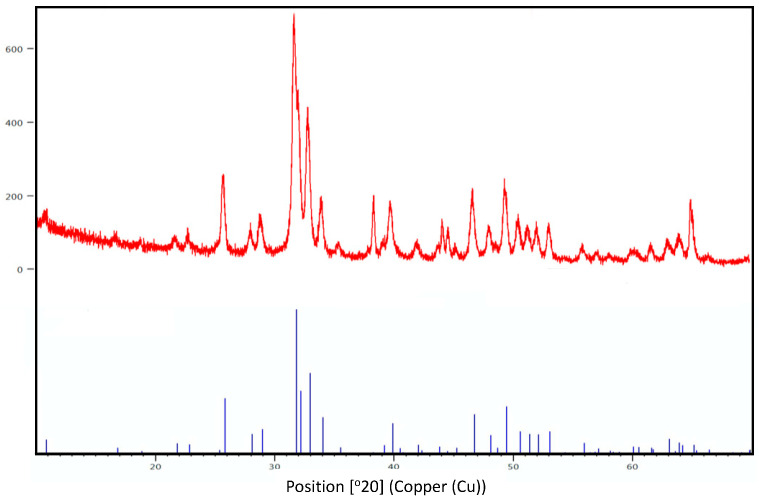
X-ray diffraction patterns of BFA (red) and JPDS pattern 01-080-3032 (blue) for fluorapatite.

The X-ray diffraction patters for BFA and pure fluorapatite (JPDS pattern 01-080-3032) are shown in Figure 3. Similar and consistent XRD patterns were observed for both BFA and pure fluorapaite found in the XRD database.

#### 3.1.3. Scanning Electron Microscopy (SEM)

**Figure 4:**
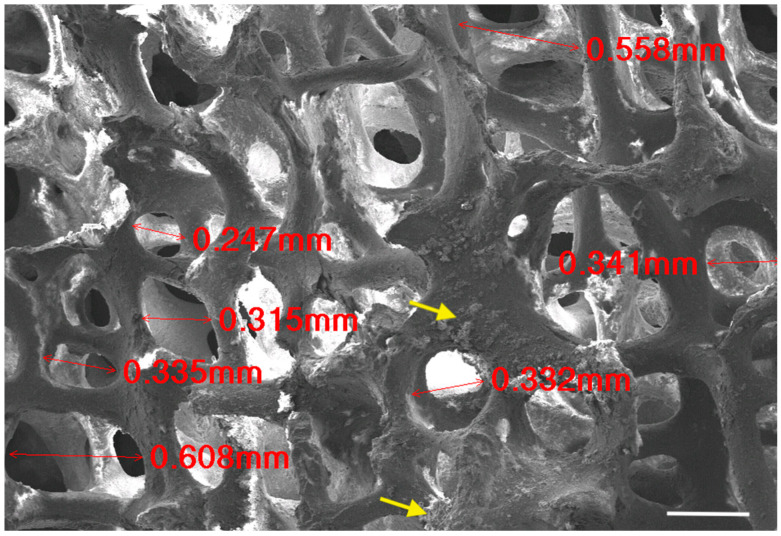
Scanning electron micrograph of the BFA scaffold. Pore sizes were measured (in red). Yellow arrows indicate the surface residues of the nano-particles deposited from the sol-gel process. Scale bar = 100 μm.

As in Figure 4, SEM analysis showed a porous and interconnected structure for the BFA scaffold. Pore sizes ranged from ~200–600 μm. Some structural deterioration and surface residues of the nano-particles from the sol-gel process were also observed.

#### 3.1.4. Energy Dispersive X-ray (EDX) and Inductively Coupled Plasma-Mass Spectrometry (ICP-MS) Analysis

**Figure 5:**
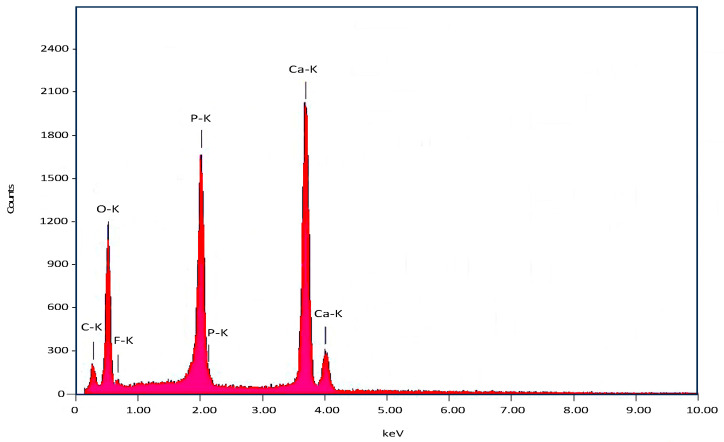
EDX analysis of the BFA scaffold.

According to the EDX spectra (Figure 5), the main inorganic constituents for BFA consist of calcium and phosphorous. The average fluoride content was approximately 1.43 wt%. The ICP-MS (Table 2) analysis showed that trace elements such as Na, Mg, K, Zn and Sr were present. Furthermore, toxic elements including cadmium (Cd) and lead (Pb) were below the detectable limits set by the ASTM standard (F1185-03) [19].

#### 3.1.5. Micro-CT Analysis

**Figure 6:**
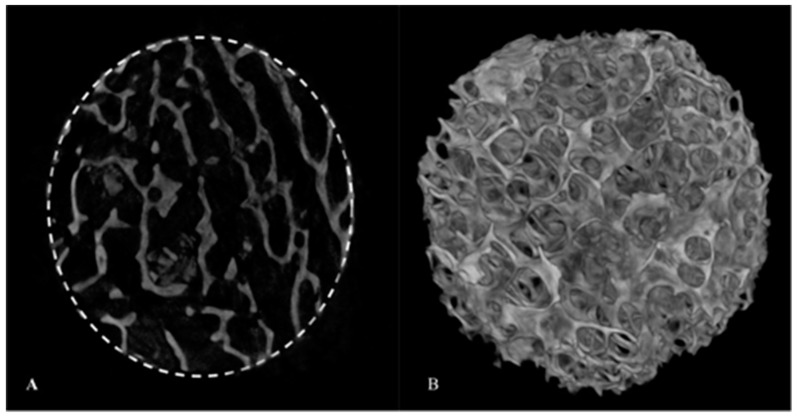
Micro-CT image of the BFA scaffold. (**A**) 2D cross-section of the BFA scaffold indicating the circular region of interest (dashed circle). (**B**) 3D image of the BFA scaffold.

The 3D structure of the BFA scaffold in Figure 6 illustrates the interconnected porous architecture. The total porosity of the BFA scaffold was calculated to be 71.62 ± 1.28%.

#### 3.1.6. Thermogravimetric Analysis (TGA) 

**Figure 7:**
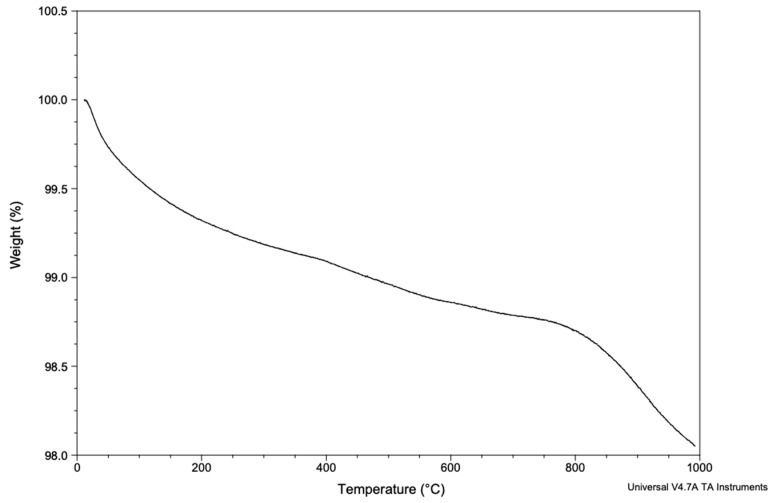
Thermogravimetric trace of BFA showing the weight loss against temperature (0–1000 °C).

The BFA sample under TGA analysis in Figure 7 exhibited a total weight loss of 1.9% when heated to 1000 °C.

### 3.2. Chemical Stability and Degradation in Simulated Body Fluid

#### 3.2.1. Chemical Stability

**Figure 8:**
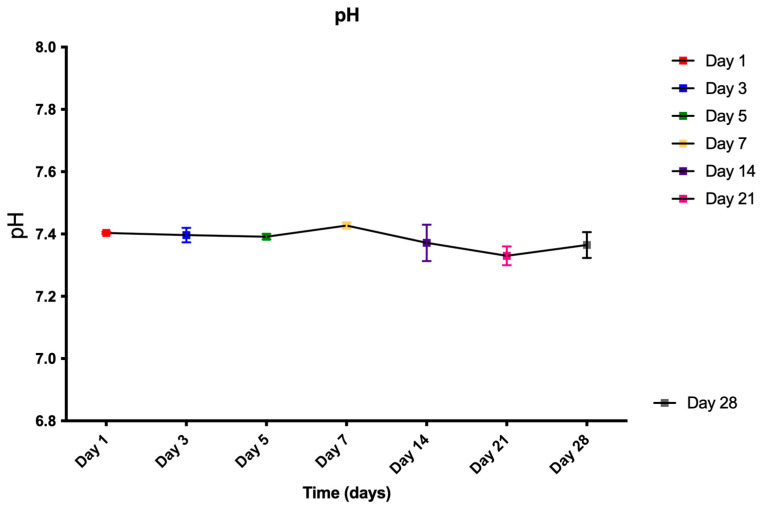
pH change of the SBF solution after each time period investigated. (n = 3). Error bars represent SE + of the mean.

The pH of the SBF solution after soaking the BFA scaffolds is shown in Figure 8. The pH fluctuated between 7.3 and 7.4 during the investigated period of 28 days. No statistically significant difference in pH was observed when analysed using a 1-way ANOVA with Tukey’s multiple comparison test (ANOVA, *p* = 0.41).

#### 3.2.2. Degradation 

**Figure 9:**
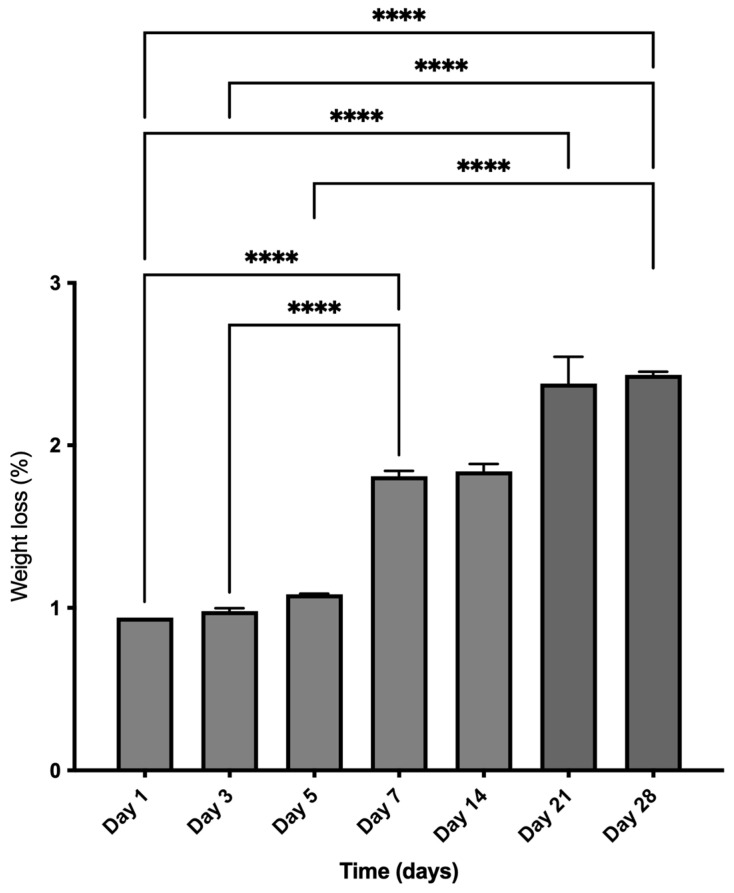
In vitro degradation of the BFA scaffolds as a function of time in SBF solution. n = 3, **** *p* < 0.0001, error bars represent SE + of the mean.

The BFA scaffold only showed minimum weight loss, losing ~2% until day 28. As shown in Figure 9, the BFA scaffold did not degrade significantly until day 5. However, following seven days, the weight loss significantly increased, peaking on day 28 (ANOVA, *p* < 0.0001).

### 3.3. Mechanical Properties

**Figure 10:**
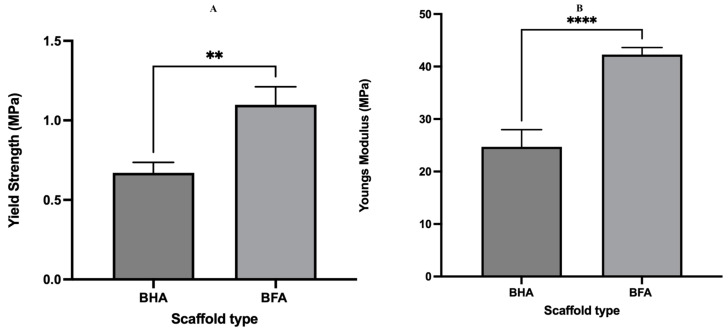
Graphical representation of the mechanical properties of the BHA and BFA scaffolds. (**A**) Young’s modulus and (**B**) Yield strength. n = 15, bars represent mean + SEM, ** *p* < 0.01, **** *p* < 0.0001.

The mechanical properties of the BHA and BFA scaffolds are represented in Figure 10. A significant increase in the yield strength (*t*-test = 0.0064) and Young’s modulus (*t*-test < 0.0001) was observed for the BFA scaffold compared to the BHA scaffold. 

### 3.4. Biocompatibility 

#### 3.4.1. Cell Viability Assay

**Figure 11:**
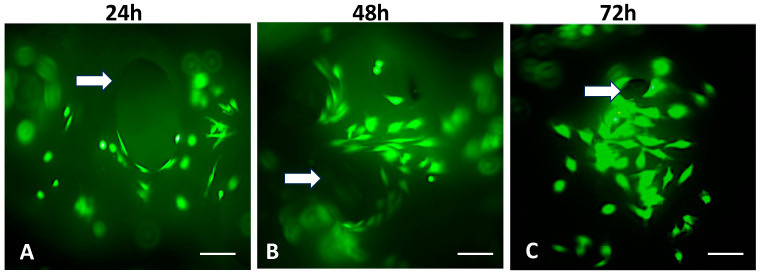
Fluorescent images illustrating the cell viability of Saos-2 cells seeded on the porous BFA scaffolds after undergoing the live/dead viability assay. White arrows represent the pores of the scaffolds (Green = live cells (calcein)). Red cells = dead cells (ethidium homodimer-1). 24 h (**A**), 48 h (**B**) and 72 h (**C**). Bar = 50 μm.

The live/dead assay shows living Saos-2 cells adhered to the BFA scaffold. Dead cells were rarely seen (Figure 11).

#### 3.4.2. Cell Proliferation Assay

**Figure 12:**
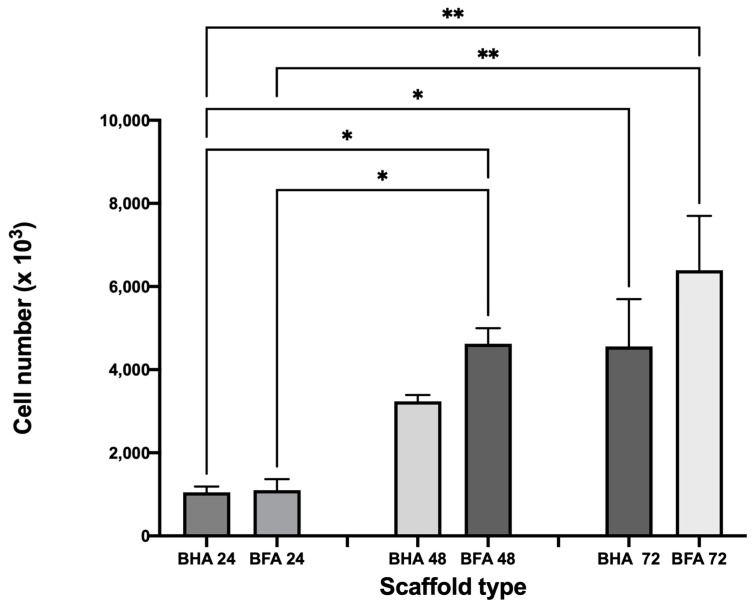
Graphical representation showing cell proliferation of Saos-2 cells after seeding onto the surface of BHA and bovine fluorapatite BFA after 24 h, 48 h and 72 h. Error bars represent the mean (±SEM) after 1-way ANOVA with Tukey’s multiple comparison test. * *p* < 0.05, ** *p* < 0.01. n = 3.

According to Figure 12, the cells proliferated on both types of scaffolds during the investigated time. However, there was no statistically significant difference in the cell proliferation between the BHA and BFA scaffolds at each time point, although the cell number on the BFA scaffold was higher at each time point than BHA.

#### 3.4.3. Alkaline Phosphatase (ALP) Assay

**Figure 13:**
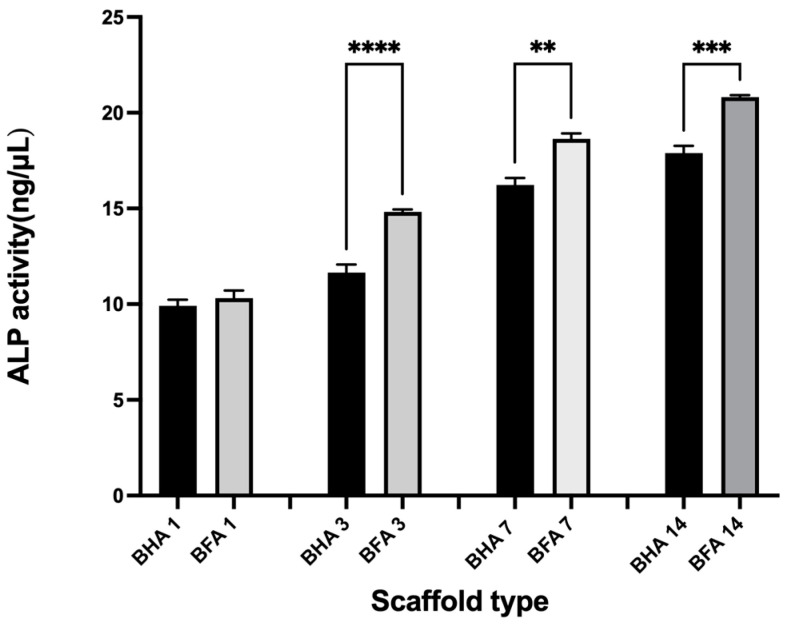
Graphical representation showing the ALP activity of Saos-2 cells after seeding on BHA and BFA after 1, 3, 7 and 14 days. Error bars represent ±SE of the mean after 1-way ANOVA with Tukey’s multiple comparison test, ** *p* < 0.01, *** *p* < 0.001, **** *p* < 0.0001, n = 3.

A significant increase in the ALP activity was observed between the BHA and BFA samples on day 3, day 7 and day 14 (Figure 13). Expression of ALP increased incrementally from day 1 to 14 for both types of scaffolds. Moreover, the BFA scaffold exhibited an increased level of ALP expression at each time point compared to the BHA scaffold.

#### 3.4.4. Immunofluorescence Assay

**Figure 14:**
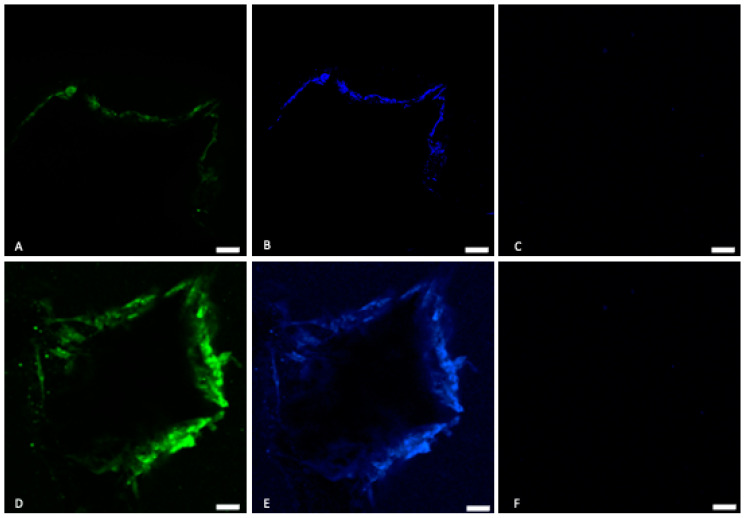
Immunofluoroscence analysis of osteonectin, on the BHA scaffold (**A**) and BFA scaffolds (**D**) After 14 days of culture. Nuclear staining/DAPI staining (blue) of the Saos-2 cells on the BHA scaffold (**B**) and SiBHA scaffold (**E**). (**C**,**F**) Control without primary antibody. Bar = 50 μm.

Immunohistochemistry results showed that the BFA scaffold was immunopositive for osteonectin after 14 days (Figure 14). A weak immunopositive result was observed for BHA. The control, which was immunostained using normal mouse IgG, revealed no staining after 14 days.

## 4. Discussion

This study aimed to substitute fluoride ions to bovine-derived hydroxyapatite (BHA) using a novel sol-gel method to produce a porous scaffold for bone tissue regeneration applications. Fluoride is an important element in our diet and essential for bone and teeth development. In dentistry, fluoride ions play a vital role in preventing dental caries as they prevent tooth demineralisation and promote remineralisation against carcinogenic bacteria [13]. Furthermore, fluoride ions have been used successfully for osteoporosis treatment because they stimulate the proliferation of osteoblastic cells [20]. As a result, fluoride-substituted synthetic hydroxyapatite (FHA) has been widely investigated over the past few decades since FHA improves synthetic HA’s biocompatibility and dissolution properties [21]. However, to date, no such studies have investigated substituting fluoride ions for mammalian-sourced hydroxyapatite.

The sol-gel method is a wet chemical method that produces a variety of products, including coatings, fibres, powders, organic/inorganic hybrids, monoliths and thin films. The sol-gel process possesses several advantages, such as homogenous molecular mixing of precursors, thereby improving the chemical homogeneity of the resulting particles; producing particles of high purity at the nano scale; utilising different chemical routes, either alkoxide or aqueous-based; and the ability to execute the reactions at low synthesis temperatures [22,23].

During the sol-gel process, an amorphous gel is created through the hydrolysis and condensation reactions of the calcium and phosphorous precursors [22,24]. Due to the reduced mixing time, calcium nitrate-tetrahydrate and ammonium hydrogen phosphate were used as calcium and phosphorous precursors, therefore benefiting the synthesis process. To improve the chemical homogeneity and to minimise rapid precipitation, a slow titration process was used during the synthesis process [25]. The addition of ammonium hydroxide catalysed the reaction between calcium nitrate tetrahydrate and ammonium hydrogen phosphate, improving the gelation process. The following reaction illustrates the formation of hydroxyapatite (HA) [22,24];

10Ca (NO_3_)_2_.4H_2_O + 6(NH_4_)_2_HPO_4_ + 8NH_4_OH → Ca_10_ (PO_4_)_6_(OH)_2_ + 20NH_4_NO_3_ + 6H_2_O

Gelation occurs when clusters grow due to the aggregation of particles, and when the clusters are linked, they form a gel [12,26]. The hydroxyl ions supplied by aqueous ammonia deprotonated the ammonium hydrogen phosphate, placing it into a more reactive state enabling it to react with the Ca precursor to form hydroxyapatite. However, the reaction would still occur without adding ammonium hydroxide, but it would take longer [16]. In this study, the experiment was conducted at 85 C. The main reason for this was that more energy is delivered into the system, increasing the reaction rate and allowing the hydroxyapatite phase to mature. In a study by Liu et al., increasing the temperature minimised the reaction time for hydroxyapatite formation [27]. In addition, conducting the experiment at 85 °C also served to evaporate any excess solvent. Cheng et al. reported that the gelation process improved when faster solvent evaporation took place [11]. 

In this study, fluoride ions were completely substituted to the hydroxyl group in the HA lattice to form fluorapatite (FA, Ca_10_(PO_4_)_6_OH_x_F_1−x_) [28]. Previous studies synthesized synthetic fluorapatite (FA) using various sol-gel routes from a variety of Ca, P and F precursors [12,28]. However, no studies have attempted substituting fluoride ions into bovine-derived hydroxyapatite (BHA). This study successfully incorporated fluoride ions into BHA using the sol-gel method to produce a bovine-derived fluorapatite (BFA) scaffold. 

Ammonium fluoride (NH_4_F) was used to prepare the fluoride sol-gel since it increased the reflux time, thus enhancing its effectiveness as a fluoride reagent [29,30]. To produce a pure FA (Ca_10_ (PO_4_)_6_F_2_) sol, the [Ca]/[P] and [P]/[F] mole ratio was adjusted to 1.67 and 3, respectively [16]. Tredwin stated that a [P]/[F] mole ratio greater than 3 would contribute to the synthesis of fluorohydroxyapatite (FHA), a partially substituted FA [31]. The BHA scaffolds were placed into the solution as soon as the white chalky colour emerged since this is where all chemical reactions take place to initiate sol-gel formation. The BHA scaffolds were mildly agitated in the saturated F sol-gel to force an ion exchange reaction. Furthermore, sintering the bones after the sol-gel process was essential to remove the remaining solvents and moisture.

The most important observation from the FTIR spectra of BFA was the absence of the hydroxyl peak observed at 3571 cm^−1^, indicating that the fluoride ions were COMPLETELY substituted into the hydroxyl group of hydroxyapatite, resulting in an ‘A type’ anionic substitution [9]. Wei et al. observed a similar result for synthetic fluorapatite [32]. In contrast, both Kim et al. and Darroudi et al. observed weak hydroxyl bands at 3572 cm^−1^ and 3570 cm^−1^, respectively, for partially substituted FA (FHA) [12,29]. In addition, the phosphate and carbonate peaks associated with HA were observed for BFA confirming the formation of fluorapatite (FA) [7,15]. The XRD patterns for BFA corresponded similarly to pure FA XRD patterns (JPDS pattern 01-080-3032), confirming the complete substitution of fluoride ions into the hydroxyl group in the hydroxyapatite lattice. Furthermore, secondary phases such as beta -tricalcium phosphate (β-TCP) (2θ ≈ 31.13°) and calcium oxide (CaO) (2θ = 37°) were not present in the XRD spectra, suggesting the phase purity of the BFA scaffolds (2θ ≈ 31.13° and 2θ = 37°). The fluoride content ranged between 1–2 wt %, and the Ca/P ratio varied between 1.67–1.79 for the BFA scaffold when analysed using EDX (Figure 5). However, the Ca/P ratio was 1.72, higher than the initial Ca/P ratio (1.67) used to synthesise the F-sol gel (Table 2) when analysed using ICPMS. The slightly higher variation of the Ca/P ratio was due to carbonate and trace amounts of sodium, magnesium and zinc (Table 2) present in bovine-derived hydroxyapatite (BHA). These vital trace elements are essential in bone metabolism, promoting osteogenesis and suppressing bone resorption [33,34].

Scanning electron micrographs confirmed a porous architecture with pores ranging from approximately ~200–600 μm (Figure 4). A porous scaffold provides a framework for osteogenic cells to migrate and proliferate, which is essential for bone formation through roles such as cellular differentiation and angiogenesis. Furthermore, a rough surface was observed for the BFA scaffold due to the deposition of particles from the sol-gel process, which is advantageous as roughness increases cellular adhesion compared to a smoother surface. The porosity results suggested that the BFA scaffold was suitable for bone tissue regeneration as the human cancellous bone has a 70% or greater porosity. Our previous study developed a BHA bone graft with a total porosity of 73.46% ± 1.08 [7], and, although some surface residues were observed, incorporating fluoride ions through the sol-gel process did not reduce the pore diameter and porosity of the BFA scaffolds compared to BHA [7]. TGA analysis showed that the BFA scaffold was thermally stable, resulting in only a total weight loss of 1.9 wt% (Figure 7). The initial weight loss (endothermic loss) observed between 50 °C and 200 °C results from the evaporation of absorbed water. The minimal weight loss (exothermic loss) between 250 °C and 600 °C results from degradation of the carbonate groups of BFA.

BFA’s highly ordered crystal structure reduced any significant weight loss, which was mainly due to the hydroxyl group being replaced by fluoride ions. In addition, substituting fluoride ions decreased the solubility of the produced BFA due to increased crystallite size and crystallinity [35].

Therefore, leaching of Ca^2+^ and PO_4_^3−^ ions from the BFA lattice was limited, which explains the negligible change in the pH value during the 28-day incubation period (Figure 7). However, post day 7, there was an increase in weight loss up to day 28 as the scaffold degraded, which is advantageous as an ideal bone grafting scaffold should degrade over time at a controlled resorption rate creating space for new bone tissue to grow [11]. In our previous study, a ~4% weight loss was observed for the BHA scaffold during the first three days and is attributed to the leaching of Ca^2+^ and PO_4_^3−^ ions from the HA lattice, which also explains the initial increase in the pH value [7]. However, the present study has a few limitations. The BFA scaffold was not placed in a proper physiological environment where the scaffold would degrade due to mechanical forces of perfusion and cellular waste products. In addition, primary enzymes such as lysozymes were absent, which would further degrade the scaffold [15].

According to these results, it was evident that the BFA scaffold exhibited superior mechanical properties compared to BHA in terms of yield strength and Young’s modulus (Figure 10). The sol-gel process used to incorporate fluoride ions into the BHA significantly improving mechanical properties. One possible reason for this could be the deposition of nanoparticles from the sol-gel process, which could be a decisive factor in improving the mechanical properties. Another reason is the structurally stable lattice structure which fluorapatite possesses [36]. 

Saos-2 cells were used to investigate the biocompatibility of the BHA and BFA scaffolds, as it is commonly used to assess the biocompatibility of biomaterials for bone regeneration applications due to their osteoblast-like properties [7,15]. The cell viability assay showed that the BFA scaffold was non-toxic and facilitated cell adherence (Figure 11). Furthermore, dead cells were present in low numbers, and cells penetrated the pores and were viable. Cellular penetration within a scaffold plays a significant role in cell infiltration and distribution, affecting the cell scaffold attachment [37,38]. Furthermore, these cells exhibit cellular protrusions (filipodia), allowing cells to extend and reach interior pores, strengthening the cell-scaffold attachment [39,40]. The cell proliferation assay illustrated that cell numbers increased up to 72 h for both BHA and BFA scaffolds, suggesting the non-toxic nature of the scaffolds allowed cells to adhere and migrate. The BFA scaffold exhibited a higher cell number than the BHA scaffold at each time point, although this result was not statistically significant. Several previous studies have shown that substituting fluoride ions into hydroxyapatite significantly increased osteoblast cell proliferation and differentiation [12,41]. The mineralisation assay showed increased ALP expression for both scaffolds during the investigated time (Figure 13). A significant increase in the ALP expression was observed for the BFA scaffold compared to BHA, which clearly showed that the BFA scaffold supported the differentiation of Saos-2 cells. The porosity of the scaffolds likely contributed to the osteoblast response, where a porous material facilitates cell differentiation and mineralization by forming cell clusters. An in vitro study conducted by Chavassieux et al. showed increased ALP activity of rat osteoblast cells when NaF was added into their drinking water compared to animals treated with no NaF addition [20]. In addition, both Cheng et al. and Kim et al. reported that incorporating fluoride ions into synthetic HA significantly increased the ALP expression level of the osteoblastic cells [12,41]. Immunohistochemical analysis for ‘*osteonectin*’ revealed a strong expression after 14 days on the BFA scaffold (Figure 14D) compared to the BHA scaffold (Figure 14A), confirming the differentiation of Saos-2 cells and suggesting that fluoride ions stimulate osteoblasts cell differentiation.

## 5. Conclusions

In this study, fluoride ions were substituted to BHA using a modified sol-gel technique to successfully produce a BFA bone graft while maintaining its porous architecture. Chemical characterisation confirmed that hydroxyl group in the HA lattice was successfully substituted by fluoride ions. The BFA scaffold possessed superior mechanical properties to BHA. The BFA scaffold was biocompatible and exhibited osteoinductive features suggesting that the scaffold constituted a suitable substrate for Saos-2 cell differentiation. The ability of the BFA to support these cellular processes may lead to new clinical options for bone tissue reconstruction therapies, such as filling surgical cavities and bone augmentation. Further in vivo experimentation is underway to evaluate the clinical feasibility of BFA in bone regeneration.

## 6. Patents

Dias GP, Ratnayake J, Niranjan R, inventors; Dias, George PJSN, assignee. ION-SUBSTITUTED BOVINE HYDROXYAPATITE FOR BONE REGENERATION. United States patent application US 17/999,882. 3 August 2023.

## Figures and Tables

**Table 1:** Constituents required to prepare F sol-gel.

Material	Ca (NO_3_)_2_.4H_2_O	(NH_4_)_2_HPO_4_	NH_4_F
RMM	236.15 gmol^−1^	132.056 gmol^−1^	37 gmol^−1^
Number of moles	0.167	0.1	0.0334
Measured weight	39.36 g	13.2 g	1.24 g

**Table 2:** Chemical composition of synthesised BFA determined by ICP-MS and SEM-EDX analysis.

Sample (mg/Kg)	Ca	P	Na	Mg	K	Zn	Sr	As	Cd	Pb
BFA	388 × 10^3^	176 × 10^3^	3.5 × 10^3^	1.47 × 10^3^	3.16 × 10^3^	0.0132 × 10^3^	0.295 × 10^3^	<0.4	<0.2	<1.8
BHA	371 × 10^3^	171 × 10^3^	4.1 × 10^3^	2.65 × 10^3^	<620	0.0189 × 10^3^	0.300 × 10^3^	<0.5	<0.25	0.81
ASTM maximum limit (F1185-03)	-	-	-	-	-	-	-	211	5	30
[Ca]/[P] molar ratio according to ICP-MS	1.72									
[Ca]/[P] molar ratio according to SEM-EDX	1.67–1.79									

## Data Availability

Data available on request.
